# Evaluation of audiovisual and auditory interventions on exam anxiety during whole-body magnetic resonance imaging in cancer patients: a quasi-experimental study

**DOI:** 10.1186/s40644-026-01060-6

**Published:** 2026-07-01

**Authors:** Lorenzo Conti, Paul Summers, Marta Mainetti, Sara Gandini, Cristiana Fodor, Giuseppe Petralia, Gabriella Pravettoni

**Affiliations:** 1 Applied Research Division for Cognitive and Psychological Science, European Institute of Oncology IRCCS, Via Ripamonti 435, 20141 Milan, Italy; 2 Division of Radiology, European Institute of Oncology IRCCS, Via Ripamonti 435, 20141 Milan, Italy; 3 Department of Experimental Oncology, European Institute of Oncology IRCCS, Via Ripamonti 435, 20141 Milan, Italy; 4 Scientific Direction, European Institute of Oncology IRCCS, Via Ripamonti 435, 20141 Milan, Italy; 5 Department of Oncology and Haemato-Oncology, University of Milan, Via Festa del Perdono 7, 20122 Milan, Italy

**Keywords:** Whole-body MRI, Audiovisual intervention, Anxiety, Cancer patients, Claustrophobia

## Abstract

**Background:**

WB-MRI is a radiation-free imaging technique widely used in oncology. Despite clinical advantages, WB-MRI can cause significant patient anxiety and claustrophobia, affecting scan completion and patient adherence. Non-invasive interventions to reduce MRI-related distress are still limited in clinical practice. This study evaluates the use of audiovisual and auditory entertainment for reducing anxiety during WB-MRI.

**Methods:**

In this prospective, quasi-experimental trial, 213 cancer patients (median age: 52.0[44–60]; female 70%) undergoing WB-MRI were equally assigned to Video (nature landscapes and sounds), Music (relaxing instrumental), or Standard (no distraction) groups. Anxiety was assessed using the State Trait Anxiety Inventory (STAI) before and after the exam. Additional measures included the Distress Thermometer, WB-MRI acceptance questionnaire, and claustrophobia ratings. Data analysis compared anxiety changes, interactions between baseline anxiety and claustrophobia, and logistic regression for anxiety reduction likelihood.

**Results:**

Baseline anxiety was comparable across groups. Anxiety reduction was greater in the Video (mean STAI = -5.57) and Music (-2.48) groups compared to the Standard (0.33) group, with the Video group significantly outperforming the Music group (*p* = 0.006). Logistic regression showed patients in Video (95%CI:3.77–68.22, *p* < 0.001) and Music (95%CI:1.47–29.8, *p* = 0.02) groups were significantly more likely to reduce anxiety than controls. Patients with severe claustrophobia and high baseline STAI exhibited pronounced improvements, particularly with Video (β = − 0.38, *p* < 0.001). The alluvial plot shows that the Video and Music groups experienced a marked reduction in the proportion of patients with severe anxiety, while this proportion slightly increased in the standard condition after the exam.

**Conclusion:**

Audiovisual and auditory entertainment significantly reduced anxiety during WB-MRI, with audiovisual showing the greatest benefit. The simultaneous involvement of multisensory distraction enhances tolerance to procedures, especially in highly anxious and claustrophobic patients. These findings advocate for the adoption of a patient-centered audiovisual system as a non-invasive, practical strategy to enhance patient experience and increase the likelihood of successful scan completion in WB-MRI practice. The results hold significant implications for optimizing patient compliance, offering tangible clinical and economic benefits, including reduced sedation requirements, prevention of long-term phobic responses, minimized motion artifacts, and improved image quality.

**Trial registration:**

Retrospectively registered on ClinicalTrials.gov NCT06332001 [first posted March 26, 2024].

**Supplementary Information:**

The online version contains supplementary material available at https://doi.org/10.1186/s40644-026-01060-6.

## Background

Whole-body magnetic resonance imaging (WB-MRI) represents an advanced, radiation-free imaging technique that enables comprehensive evaluation of the entire body. Its application is well established in oncological practice and supported by international guidelines, such as MET-RADS-P, ONCO-RADS, and MY-RADS, for the standardized assessment of various malignancies [1–3]. WB-MRI is particularly effective for detecting both bone and soft-tissue lesions, providing accurate tumor staging and supporting optimal therapeutic monitoring [3–6].

Although WB-MRI is considered a non-invasive and painless procedure, it may be uncomfortable in specific medical contexts (e.g., bone malignancies); therefore, adequate patient preparation is essential to ensure its acceptability and smooth integration into the clinical workflow. Several psychological factors may influence patient acceptance of WB-MRI, potentially affecting the efficiency of the diagnostic process [7, 8]. Adult patients often experience significant anxiety, fear, or claustrophobia during the examination, sometimes requiring sedation [7, 9]. Evidence from MRI studies suggests that up to 37% of adults may experience severe distress both before and during the examination [6, 8, 10]; although these findings derive from studies involving different MRI procedures, they highlight a broader concern regarding patient anxiety during MRI imaging. The main source of anxiety is usually the confinement in a narrow space for extended periods; however, additional stressors such as worry about the diagnostic outcome, loud scanner noise, vibrations, environmental isolation, and immobilization may further exacerbate patient discomfort and increase anxiety [10].

These inconveniences may lead up to 5% of patients to prematurely interrupt scanning [11, 12]. Furthermore, post-scan anxiety can contribute to the development of MRI-related fear or long-term phobia [13, 14]. Even when scanning is completed, coping with high levels of distress may result in motion that compromises image quality and reduces diagnostic accuracy [13, 15]. This is particularly evident for patients with high anxiety or severe claustrophobia, where an exhaustive education preparation regarding the WB-procedure may not be sufficient. Therefore, targeted strategies such as resource-focused counseling, relaxation training, or distraction from disturbing stimuli should be implemented to reduce patient distress during WB-MRI scans [6, 8].

The difficulties experienced by patients, combined with the limited availability of WB-MRI, represent significant barriers to care for the general population [16]. Consequently, data on effective strategies to improve patient experience during the WB-MRI examination are scarce. Therefore, identifying interventions that facilitate completion of diagnostic WB-MRI is crucial, as minimizing patient discomfort may enhance participation in screening programs and promote early diagnosis.

These considerations have fostered interest in strategies to improve the MRI experience and ensure the optimal management of this clinical examination. The purpose of this prospective study is to evaluate the acceptability and tolerability of WB-MRI examinations when audiovisual strategies are provided. These approaches are compared to the conventional standard of care to determine their efficacy in reducing patient anxiety and improving the WB-MRI experience.

## Methods

### Participants and study design

This study enrolled both outpatient cancer patients undergoing WB-MRI examinations for detection, staging, or therapy monitoring, as well as individuals participating in cancer screening at the Hospitalization and Healthcare (IRCCS) European Institute of Oncology (IEO), in Milan. Subjects who had scheduled a WB-MRI exam were contacted and invited to participate in the study between January 2023 and June 2025. Individuals who expressed interest were interviewed at the IRCCS IEO on the day of their examination to complete the informed consent process. Eligibility criteria included absence of psychiatric conditions or other disorders that could impede participation, as well as no contraindications to MRI (e.g., first-trimester pregnancy or certain metal implants).

All participants received detailed instructions from experienced healthcare providers regarding the procedure. They were informed that the examination would last approximately 45 min and would require breath-holding at several points. In addition, explanatory panels summarizing the key instructions of the WB-MRI procedure were displayed in the waiting area.

**Fig. 1:**
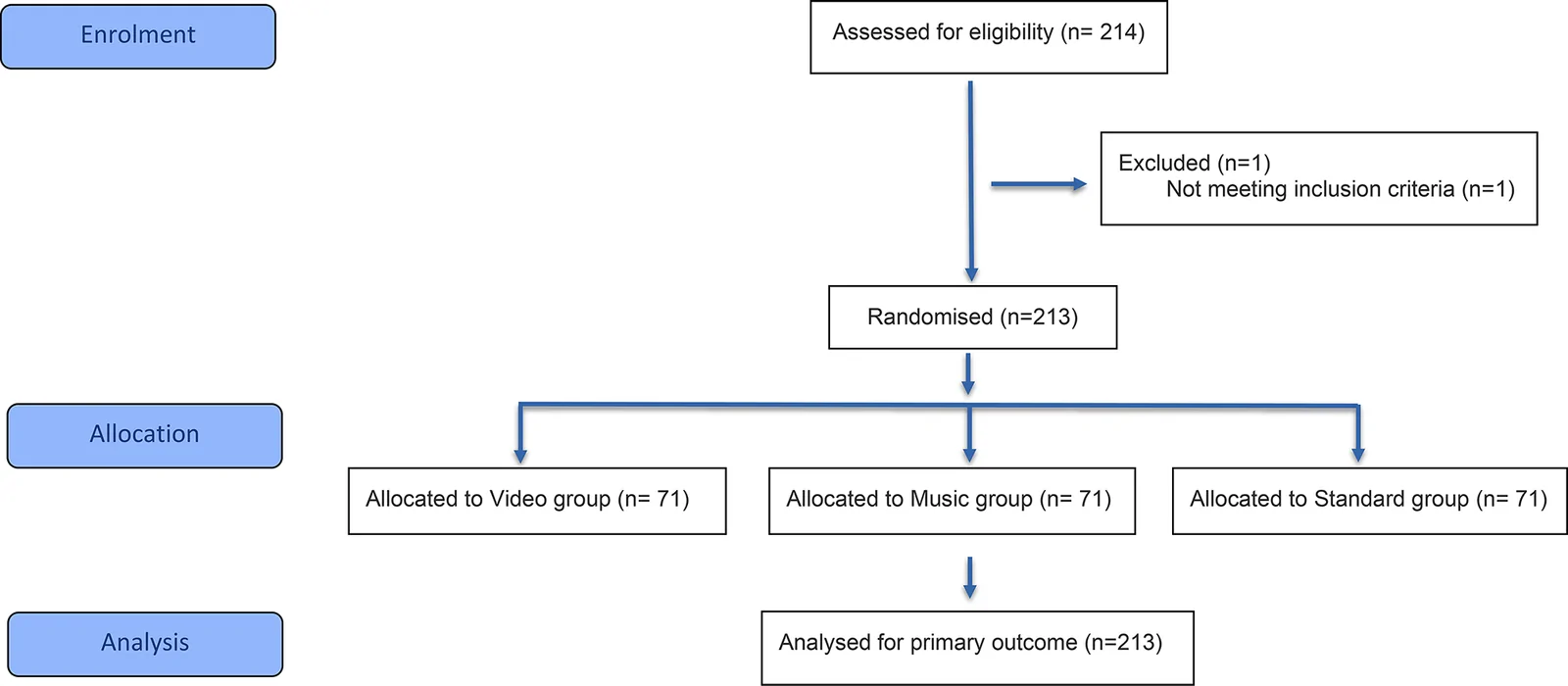
CONSORT 2025 flow diagram. Flow diagram of the progress through the phases of a randomised trial of three groups (that is, enrolment, intervention allocation, and data analysis)

In this quasi-experimental design, a total of 213 participants were randomly and equally assigned to one of the three parallel groups: Music arm, which received musical entertainment during the examination; Video arm, which received visual entertainment combined with relaxing music; and Standard control group, which underwent the standard care procedure **(see** Fig. 1**)**. All the participants received frequent communicative interactions with healthcare professionals during the WB-MRI exam to provide ongoing feedback.

### WB-MRI procedure

The WB-MRI protocol was compliant with the ONCO-RADS guidelines [3] and included the following sequences: axial spin-echo echoplanar diffusion-weighted, gradient echo T1-weighted dual-echo Dixon, and half acquisition turbo spin echo T2-weighted images acquired from the skull base to mid-thigh; sagittal fat-saturated T2-weighted images of the spine; axial fluid-attenuated inversion recovery (FLAIR) T2-weighted image of the brain; and axial short echo time gradient-echo proton density-weighted images of the lungs. The entire protocol lasts approximately 40 min. The two MRI scanners employed in this study operated at 1.5 and 3 T (Avanto Fit and Skyra, respectively, Siemens Healthineers, Erlangen, Germany). Anatomy-specific phased-array surface coils were used to optimize image quality for all body regions examined.

### Psychological assessment

The psychological assessment was performed at two time points: immediately prior to the WB-MRI exam (T0) and immediately following its completion (T1). At T0, each of the participants met the psychologist and filled out the following questionnaires:

- The pre-exam section of the WB-MRI acceptance questionnaire [17] to assess subjects’ confidence, concerns, and perceived utility of the WB-MRI exam.
- The Distress Thermometer (DT) [18, 19] to rate the perceived distress and the sources of discomfort experienced over the past week.
- The State-Trait Anxiety Inventory subscale (STAI-Y) [20, 21] to assess the level of anxiety at the time of completion.

Participants were also asked to rate their level of claustrophobia, classifying it as absent, mild, or severe.

At T1, a further psychological assessment was administered. Specifically, all participants completed:

- The post-exam section of the WB-MRI acceptance questionnaire [17] to evaluate the experience with the procedure and the acceptability of the WB-MRI based on the interventions carried out.
- The MRI-anxiety questionnaire (MRI-AQ) [10], to investigate patients’ anxiety during MRI examinations.
- The State–Trait Anxiety Inventory subscale (STAI-Y) [20, 21].

### Multimodal intervention

**Fig. 2:**
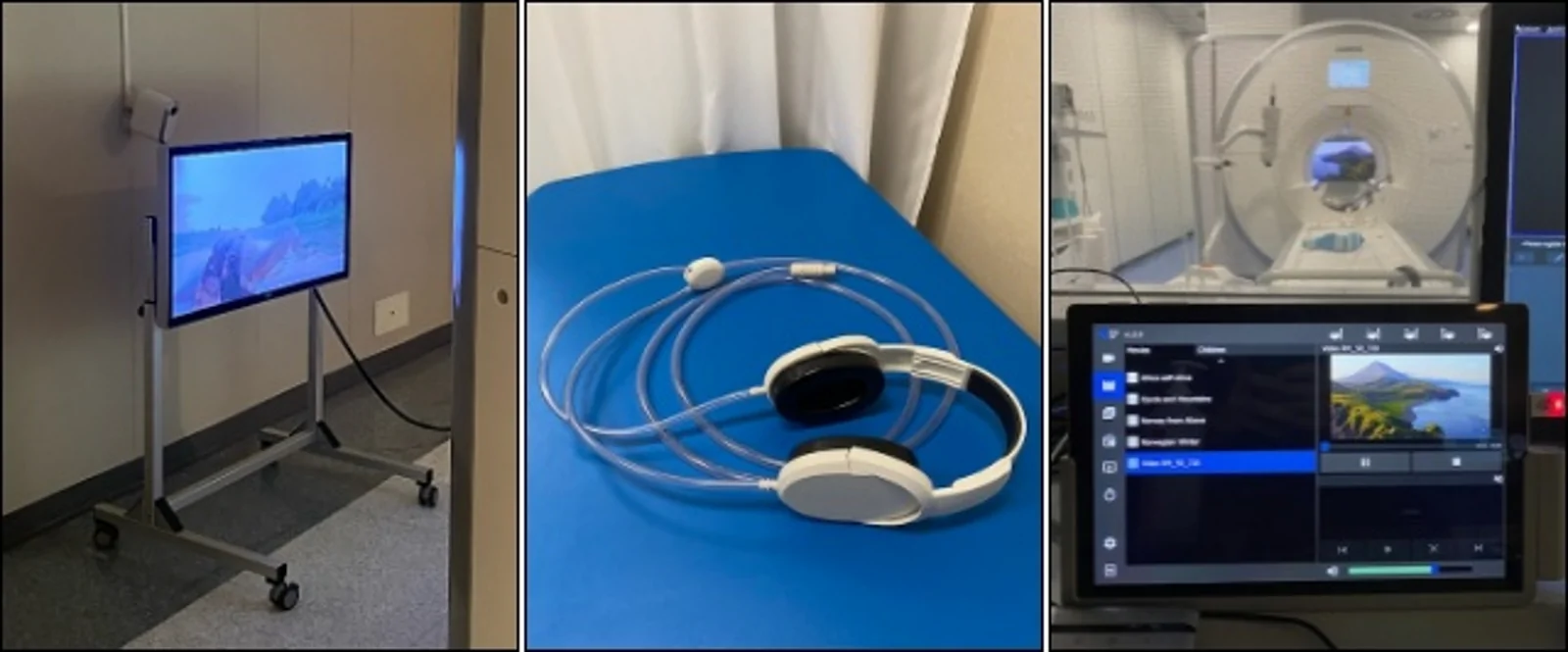
Nordic NeuroLab audiovisual system (monitor, headphones and remote control device)

In this study, the Nordic NeuroLab audiovisual system was adopted during the WB-MRI examination to provide patients with multi-sensory entertainment (See Fig. 2). This system comprises three main components:

- The In-room Viewing Monitor (Nordic Neurolab, Fig. 2), a 40” high-definition LCD designed to operate safely within the MRI environment, compliant with the Medical Device Regulation (EU) 2017/745.
- Headphones (Siemens Healthineers, Fig. 2), approved with EU-CE mark and compatible with standard head and body coils. These headphones provide noise dampening, allow transmission of audio content such as music, and facilitate communication between the patient and the healthcare provider.
- Nordic Comfort Player Device (Nordic Neurolab, Fig. 2), a touchscreen control system used from the MRI control room to manage and synchronize audio-visual content throughout the procedure.

Participants used these devices according to their assigned experimental group.

Individuals in the Standard arm underwent the WB-MRI under conventional conditions. They were positioned comfortably on the scanner bed with coils placed over the body, and provided with headphones to attenuate scanner noise as well as to maintain communication with the technician.

Participants in the Music arm had the same setup but listened to relaxing instrumental music via headphones during the WB-MRI scan. The music selection primarily featured piano and acoustic guitar pieces without vocals.

**Fig. 3:**
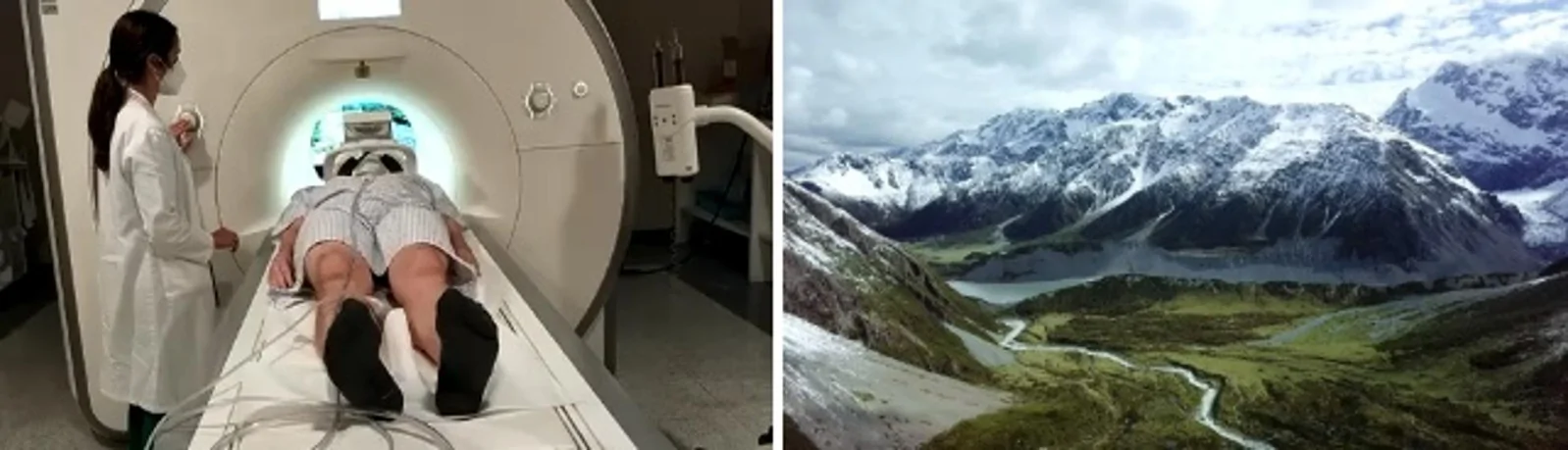
Patient preparation for WB-MRI (*left*) and an example of a natural scenario included in the video projected during the WB-MRI examination (*right*)

Finally, participants in the Video arm received combined audiovisual stimulation through a monitor viewed via mirror goggles and headphones. The audiovisual content consisted of videos depicting natural landscapes and wildlife, accompanied by relaxing nature sounds. Selected scenarios included aerial views and smooth transitions through seascape and mountainous environments across different seasons, with animals filmed in their natural habitats (see Fig. 3).

### Statistical procedures

Continuous variables were summarized using the median and interquartile range (IQR), while categorical variables were described using absolute and relative frequencies. Differences across the three intervention arms were assessed using the Kruskal–Wallis test for continuous variables and the chi-squared test for categorical variables. Differences between patients were also investigated based on the indication for the exam (screening vs. diagnosis) and ongoing oncological treatment.

To assess the interventions’ efficacy on anxiety levels, linear regression models were fitted to the absolute change in STAI score, adjusting for baseline STAI. The assumption of normally distributed residuals was assessed, and scatterplots were used to explore potential interactions between continuous covariates and categorical variables with the outcome.

Differences among study arms in terms of anxiety rates were further explored using independent contrasts within regression models, including both the intervention arm and the baseline STAI score. Initially, participants assigned to the Video and Music arms were grouped and compared to those in the Standard arm, followed by a direct comparison of the Video versus Music arms. Additional exploratory analysis considered the impact of relevant covariates (e.g., claustrophobia perceived during MRI) and interaction terms. The adjustment for baseline STAI ensured control for inter-individual variability in pre-exam anxiety, preventing confounding by participants’ initial anxiety states. Models reported included only variables statistically significant in univariate analyses.

For added clinical relevance, the study endpoint was redefined by examining categorical improvement in anxiety levels. Patients were classified as having Low anxiety levels if their STAI score was < 37, Moderate if their score was between 38 and 44, and Severe anxiety with a STAI score > 45 [22]. Transitions between anxiety categories were visualized using an alluvial plot.

The analyses were conducted using R (version 4.4.2). A two-sided significance level of 0.05 was used for all statistical tests.

## Results

### Descriptive characteristics of the population by arms and time

A total of 213 patients were recruited for the study and were split equally among the intervention arms: Standard, Music, and Video, each with 71 subjects. Table 1 summarizes the socio-demographic characteristics of the participants. The overall median age was 52 years (IQR: 44–60; range: 26–79) and was similarly distributed among experimental groups. The female-to-male ratio (2.38: 1) was identical across study arms. Regarding the educational level, 116 participants (54.5%) had a university-level education, whereas 97 (45.5%) had a high school or lower degree. Focusing on the clinical information and the MRI characteristics (see Table 1), 153 participants had previously undergone an MRI, whereas 60 underwent the WB-exam for the first time. MRI was performed for screening purposes in 40 patients (18.8%, *p* = 0.81). Among individuals enrolled in the study, the majority were patients with existing cancer diagnoses (*n* = 173), of whom 85 (49.1%) were undergoing oncological treatment, and 45 (21.1%) had at least one metastasis (see Table 1).

**Table 1:** Baseline clinical and socio-demographic characteristics of participants

Characteristic		Study Arm				*p*-value
		Standard	Video	Music	Overall	
Covariate		Median (IQR) or *N* (%)				
Age (years)		54 (44;59.5)	52 (42;61)	52 (46.5;59)	52.0 (44;60)	> 0.99
Gender	*Female*	50 (70.4%)	50 (70.4%)	50 (70.4%)	150 (70.4%)	> 0.99
	*Male*	21 (29.6%)	21 (29.6%)	21 (29.6%)	63 (29.6%)	
Education	*High (> 13y)*	41 (57.7%)	37 (52.1%)	38 (53.5%)	116 (54.5%)	0.78
	*Low (< 13 y)*	30 (42.3%)	34 (47.9%)	33 (46.5%)	97 (45.5%)	
Screening	*Yes*	12 (16.9%)	13 (18.3%)	15 (21.1%)	40 (18.8%)	0.80
	*No*	59 (83.1%)	58 (81.7%)	56 (78.9%)	173 (81.2%)	
Previous DWB-MRI	*Yes*	48 (67.6%)	51 (71.8%)	54 (76.1%)	153 (71.8%)	0.54
	*No*	23 (32.4%)	20 (28.2%)	17 (23.9%)	60 (28.2%)	
Metastasis	*Yes*	17 (23.9%)	17 (23.9%)	11 (15.5%)	45 (21.1%)	0.42
	*No*	42 (59.1%)	41 (57.7%)	45 (63.4%)	128 (60.1%)	
Cancer treatment	*Yes*	29 (40.8%)	29 (40.8%)	30 (42.3%)	85 (39.9%)	0.88
	*No*	30 (42.3%)	29 (40.8%)	26 (36.6%)	88 (41.3%)	

There were no differences in emotional distress and pre-exam anxiety levels among the participants in this study (Table 2). Regarding the perception of claustrophobia, 127 patients (59.6%) did not experience any claustrophobia, while 47 patients (22.1%) reported mild claustrophobia, and 39 (18.3%) reported severe claustrophobia (Table 2). However, there was no statistically significant difference (*p* = 0.14) in the distribution of perceived claustrophobia categories between the different experimental groups.

Conversely, both the median MRI-AQ score (*p* = 0.01) and the median post-examination STAI score (*p* = 0.04) differed statistically between the different experimental groups (Table 2).

**Table 2:** Emotional distress and participants’ characteristics during and after WB-MRI exam

ARM		Standard	Video	Music	Overall	*p*-value
Covariate		Median (IQR) or *N* (%)				
Distress Thermometer		2.0 (1.0;4.5)	2.0 (0.0;4.5)	3.0 (1.0;4.5)	2.0 (1.0;5.0)	0.56
Perceived Claustrophobia	*Absent*	49 (69.0%)	34 (47.9%)	44 (62.0%)	127 (59.6%)	0.14
	*Mild*	12 (16.9%)	20 (28.2%)	15 (21.1%)	47 (22.1%)	
	*Severe*	10 (14.1%)	17 (23.9%)	12 (16.9%)	39 (18.3%)	
STAI score pre-exam		34.0 (29.0;40.0)	36.0 (30.5;46.5)	33.0 (28.5;40.0)	34.0 (29.0;43.0)	0.13
STAI pre-exam category	*Low*	49 (69.0%)	36 (50.7%)	48 (67.6%)	133 (62.4%)	**0.046**
	*Moderate*	12 (16.9%)	11 (15.5%)	7.0 (9.9%)	30 (14.1%)	
	*High*	10 (14.1%)	24 (33.8%)	16 (22.5%)	50 (23.5%)	
STAI score post-exam		34.0 (29.0;41.0)	30.0 (26.0;38.0)	32.0 (27.5;38.0)	32.0 (28.0;40.0)	**0.04**
STAI post-exam category	*Low*	48 (67.6%)	51 (71.8%)	52 (73.2%)	151 (70.8%)	0.84
	*Moderate*	10 (14.1%)	11 (15.5%)	10 (14.1%)	31 (14.6%)	
	*High*	13 (18.3%)	9 (12.7%)	9 (12.7%)	31 (14.6%)	
Δ STAI score (post-pre)		0 (-1.0;1.0)	-5 (-10.0; -2.0)	-2 (-4.0;0.0)	-1.0 (-5.0;0.0)	**< 0.001**
MRI-AQ		22.0 (18.0;28.5)	19.0 (16.0;25.0)	20.0 (16.0;24.0)	20.0 (16.0;26.0)	**0.01**

**Fig. 4:**
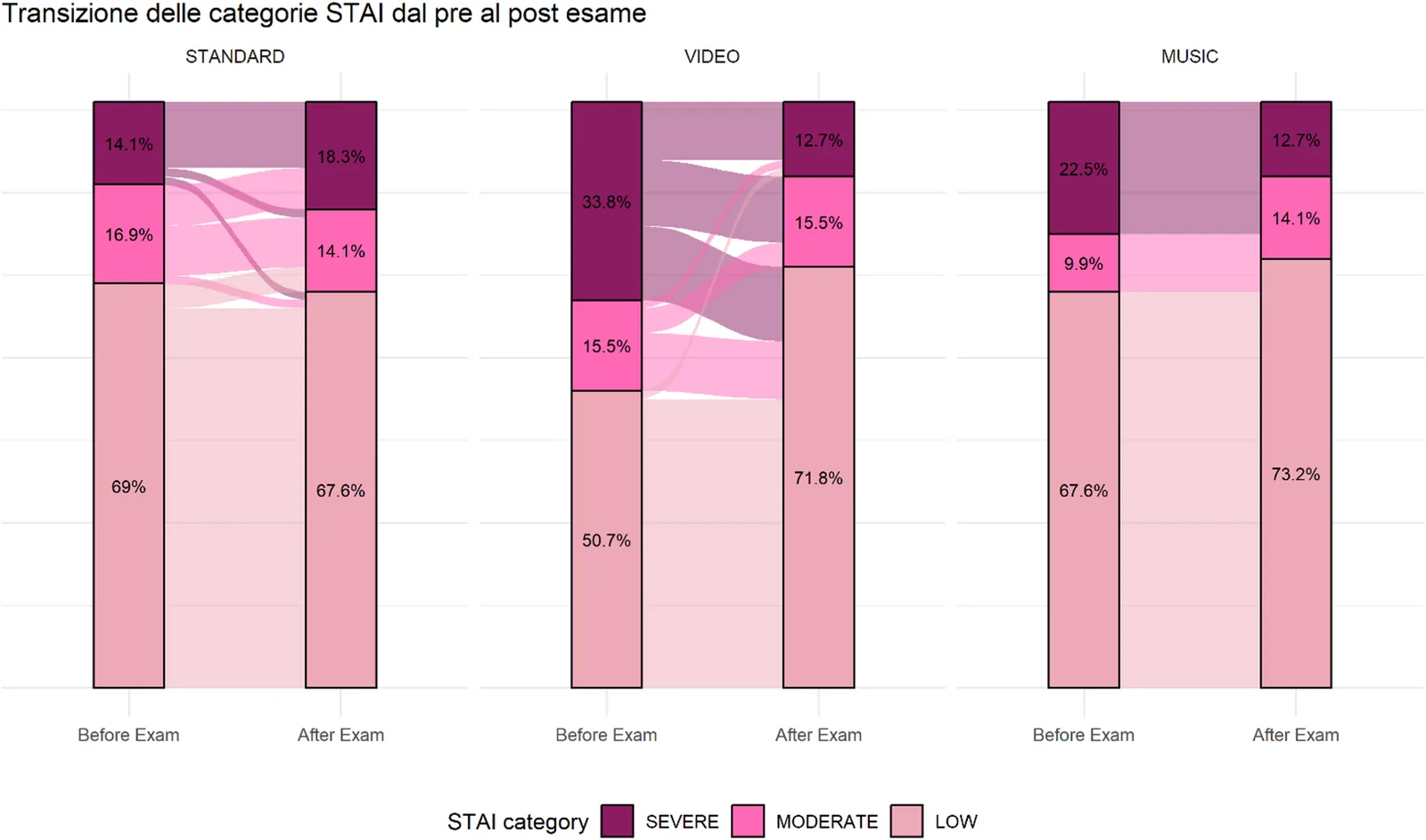
Transition of patients in STAI categories from before to after the WB-MRI, divided by arm

The alluvial plot in Fig. 4 illustrates the distribution of STAI score categories, measured both before and after the exam, across the different intervention arms (Table 2). The Video arm reported a substantial reduction in the proportion of patients experiencing severe anxiety, which dropped from 33.8% before the exam to 12.7% afterward. Similarly, the percentage of individuals in the Music arm reporting severe anxiety dropped from 22.5% before the exam to 12.7% afterward. The only condition where the proportion of patients reporting severe anxiety increased post-exam was the standard condition, moving from 14.1% to 18.3%.

### Main effects: differences in STAI among arms

**Fig. 5:**
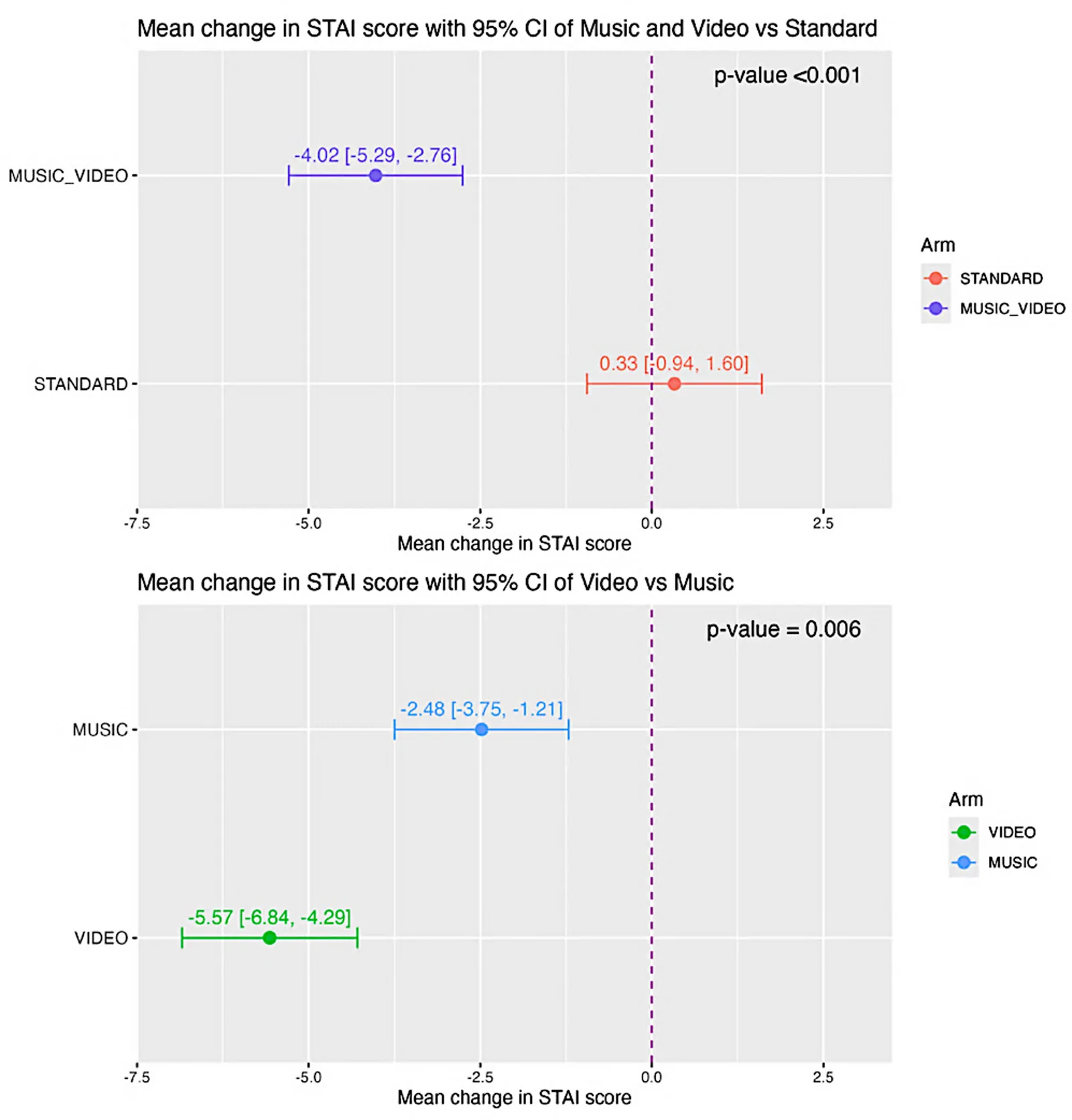
Changes in STAI score between experimental arms, adjusted for the baseline STAI score

As seen in Fig. 5, the adjusted mean change in STAI score for the combined Video-Music interventions was − 4.02 (95% Confidence Interval [CI]: -5.29 to -2.76), indicating a substantial reduction in anxiety levels following the intervention. In contrast, the Standard arm exhibited a mean change of 0.33 (95% CI: -0.94 to 1.60). The non-overlapping confidence intervals between these groups provide evidence of statistically significant group differences (*p* < 0.001).

Comparing the two intervention groups, the adjusted mean change in STAI score for the Music arm is -2.48 (95% CI: -3.75 to -1.21), while the Video arm showed a more pronounced decrease with a mean change of -5.57 (95% CI: -6.84 to -4.29). The difference between these intervention arms is statistically significant (*p* = 0.006).

### Linear models

**Fig. 6:**
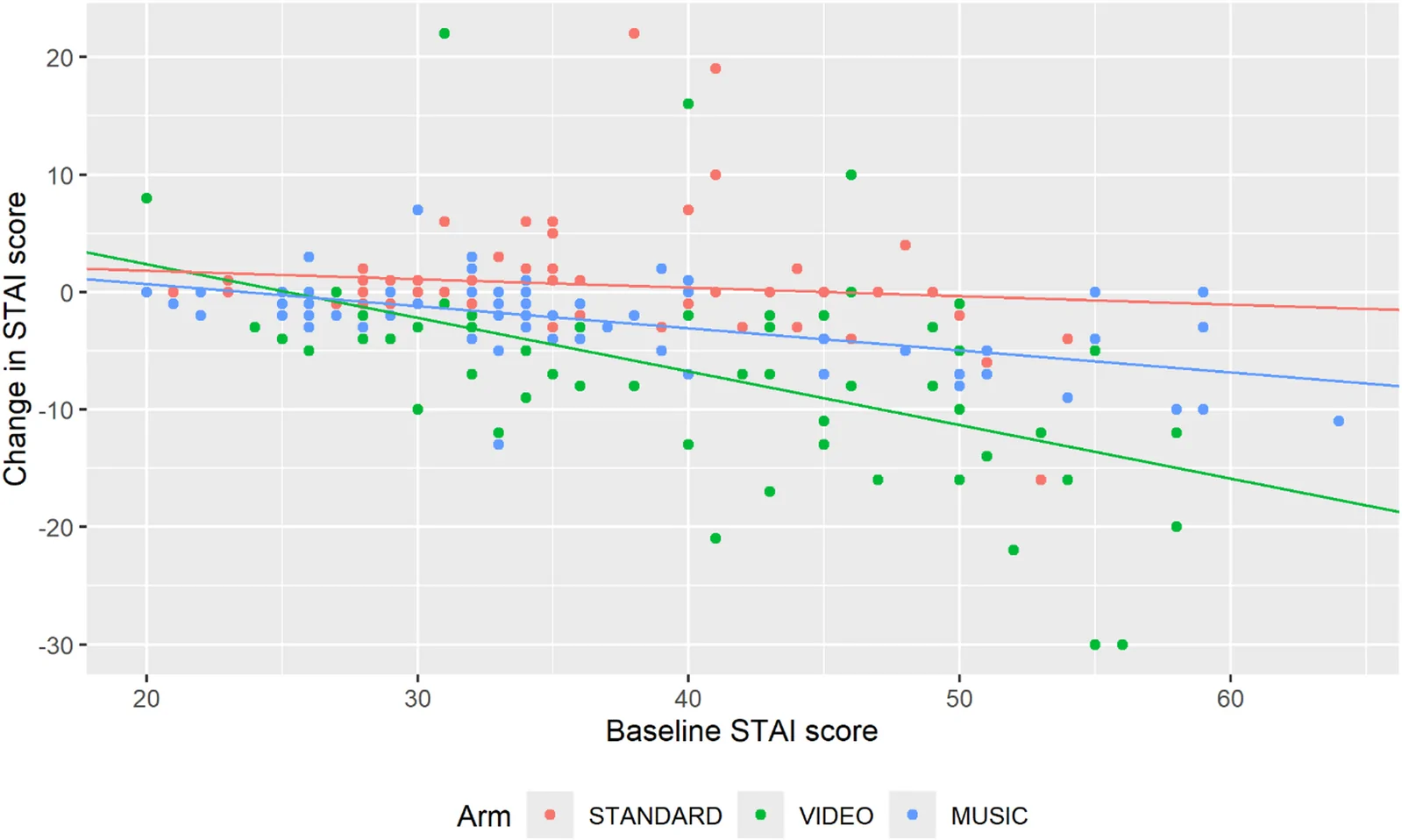
Scatterplot of the change in STAI scores against baseline STAI score with linear estimates by experimental arm

The estimated coefficients derived from a linear regression model that incorporates the effects of the different intervention arms, the baseline STAI score, and their interaction on post-exam STAI score are given in Table 3. The analysis revealed a significant interaction between baseline STAI scores and the Video intervention arm (β = -0.38, *p* < 0.001, CI: -0.58, -0.18). Specifically, patients in the Video arm exhibited a greater reduction in post-exam STAI score with higher baseline scores. This trend contrasts with the Standard condition, where no such interaction effect was observed. This dependence of STAI decrease on baseline anxiety is seen graphically in Fig. 6.

**Table 3:** Coefficients of linear model adjusted by arm and baseline STAI score

Variable	Estimate (Beta)	Lower CI 95%	Upper CI 95%	*p*
Intercept	3.27	-2.50	9.03	0.27
Video vs. Standard	8.21	0.70	15.7	**0.032**
Music vs. Standard	1.15	-6.08	8.38	0.75
Baseline STAI score	-0.07	-0.23	0.088	0.38
Interaction Video*STAI	-0.38	-0.58	-0.18	**< 0.001**
Interaction Music*STAI	-0.12	-0.31	0.083	0.25

The linear regression model also considers the perceived claustrophobia during MRI (Table 4). First, the video intervention and its interaction with baseline anxiety levels remain a key finding (*p* < 0.001), consistent with the previous model. In particular, the coefficient for the video intervention remains significant and close to its value in the earlier model (-0.37 vs. -0.38). Second, the model reveals that patients who experienced severe claustrophobia showed a significant reduction in their STAI scores, with a decrease of -0.36 points for each unit increase in their baseline STAI score (ϐ= -0.36, *p* = 0.004, CI: -0.60, -0.11).

**Table 4:** Coefficients of the linear model adjusted by Arm, claustrophobia, and baseline STAI score

Variable	Estimate (Beta)	Lower CI 95%	Upper CI 95%	*p*-value
Intercept	0.48	-5.81	6.76	0.88
Video vs. Standard	7.61	0.12	15.10	**0.046**
Music vs. Standard	1.62	-5.59	8.82	0.66
STAI score pre-exam	0.01	-0.17	0.20	0.87
Mild vs. Absent Claustrophobia	1.21	-6.69	9.11	0.76
Severe vs. Absent Claustrophobia	13.88	3.12	25.64	**0.011**
Video*STAI	-0.37	-0.57	-0.17	**< 0.001**
Music*STAI	-0.13	-0.33	0.064	0.19
Mild claustrophobia*STAI	-0.014	-0.22	0.19	0.89
Severe claustrophobia*STAI	-0.36	-0.60	-0.11	**0.004**

* Indicates interaction term

**Fig. 7:**
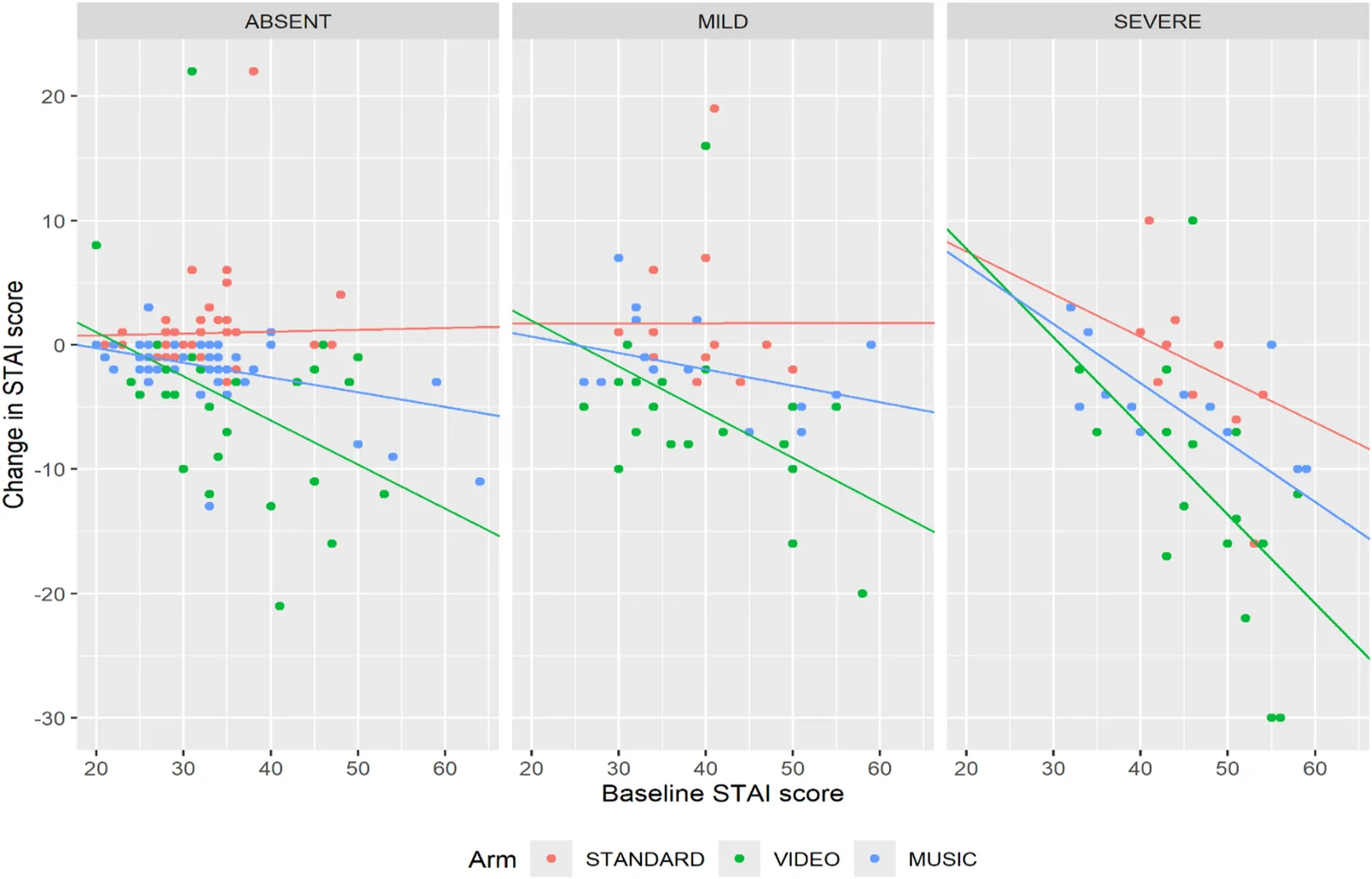
Scatterplot of the change in STAI scores against baseline STAI score, stratified by claustrophobia, with linear approximation by experimental arm. The interaction between severe claustrophobia and the STAI score can be seen in the differing slopes of the linear regression lines across the different categories of claustrophobia severity

Figure 7 visually depicts the relationship between claustrophobia severity and anxiety during the MRI, while also highlighting how these relationships differ across the levels of perceived claustrophobia. The differing slopes suggest that the effect of baseline anxiety on changes in STAI score is not uniform across all claustrophobia categories. The plot underscores how baseline anxiety is associated with differential changes in STAI scores, depending on the level of claustrophobia experienced during the MRI.

Furthermore, the interaction is strongest for the Video arm, but exists to a lesser extent for the Music arm. The lines representing the Video arm demonstrate a distinct pattern compared to other interventions, suggesting that the video may be more effective for individuals with higher baseline anxiety, as reflected in the changes in their STAI scores.

### Analysis of improvement from moderate to severe anxiety: Linear models with binary outcomes

The logistic regression model developed to assess the association between intervention arms and the likelihood of improvement in anxiety levels, as defined by a transition from moderate/severe to a lower anxiety category, showed that both audiovisual interventions significantly increase the odds of anxiety improvement when compared to the control group. In particular, the Video arm demonstrated a striking effect (OR = 13.8, *p* < 0.001, CI: 3.77, 68.22), and the Music arm was associated with an odds ratio of 5.81 (OR = 5.81, *p* = 0.02, CI: 1.47, 29.8).

## Discussion

Over the past decade, WB-MRI has gained significant clinical importance due to its extensive anatomical coverage without ionizing radiation exposure [4, 6, 23]. Its widespread adoption in oncology, supported by international guidelines, such as MET-RADS-P, ONCO-RADS, and MY-RADS, has established WB-MRI as a valuable tool for disease detection and management [1–3, 6]. Despite being a painless and non-invasive procedure, WB-MRI can induce distress and difficulty in completing the examination due to physical confinement, loud noise, and immobilization requirements, as observed in general MRI procedures [8]. While various non-invasive strategies, such as cognitive-behavioural techniques and relaxation exercises, have been proposed to alleviate patient distress, robust evidence supporting their widespread clinical adoption is still lacking. To address the need to identify effective interventions, our prospective study aimed to assess the acceptability and tolerability of WB-MRI enhanced with adjunctive audiovisual interventions to reduce anxiety and improve patient adherence, compared to the standard imaging procedure.

Participants were randomly allocated to one of the three experimental groups (i.e., video, music, and standard), and baseline assessment confirmed no significant differences in pre-MRI anxiety and distress, thus minimizing potential confounding factors. Following the WB-MRI, however, anxiety levels showed significant differences among the experimental groups, highlighting a notable effect of the type of intervention on the patient’s perception of the MRI experience.

The results underscore the superior efficacy of audiovisual stimuli over conventional methods in alleviating participant anxiety, as reflected by the change in STAI scores measured before and after the DWB-MRI procedure. This is consistent with broader research on multisensory integration, which emphasizes its fundamental role in modulating distress and patients’ discomfort during medical procedures [24]. The principle widely applied across diverse clinical settings to enhance the patient experience is that of distraction [25]. As an emotion-focused coping strategy, it redirects attention from aversive stimuli towards pleasant or engaging sensory inputs, thereby alleviating stress and anxiety [26, 27]. This neural mechanism underlies the strategies adopted in our study, combining inputs from multiple sensory modalities to shape perception, decision-making, and behavior [27].

A more detailed analysis comparing the two intervention groups (i.e., Video vs. Music) revealed that both modalities effectively reduced anxiety but significantly differ in the magnitude of their effect. The Video group achieved a greater mean reduction in anxiety scores (-5.57 points) compared to the Music group (-2.48 points), with this difference reaching statistical significance (*p* = 0.006). These findings suggest that the video intervention may exert a stronger modularity effect on the neural mechanisms underlying anxiety during WB-MRI.

Active engagement with music during MRI examinations has been shown to significantly reduce anxiety and improve patient tolerance beyond what is achieved by passive noise reduction methods like earplugs or standard headphones [28, 29]. Nevertheless, the WB-MRI examination is itself very noisy, and may interfere with or limit the ability to perceive Music throughout the examination. For this reason, the addition of visual stimuli appears to amplify the immersive distraction effect, thereby further improving patients’ comfort by altering their perception of MRI scanner noise and the overall procedural experience [30]. Encouraging evidence of the greater immersive potential of multimodal stimulation has also been reported in other clinical populations and medical procedures. Kaulfuss et al. (2025) found that audiovisual entertainment, compared to audio distraction alone and a control condition, significantly enhanced patients’ subjective perception of the duration of perineal prostate examination [31]. Similarly, Chirico et al. (2020) reported that both visual (e.g., virtual reality) and auditory (e.g., music therapy) interventions effectively reduced anxiety and improved mood in breast cancer patients undergoing chemotherapy, relative to standard care [25]. These findings underscore the broad applicability of sensory-based distraction strategies across various therapeutic contexts.

The superior effectiveness of audiovisual interventions can also be attributed to the intrinsic characteristics of the stimuli. Evidence from studies utilizing natural landscape videos and sounds demonstrates the powerful calming effects of nature during stressful medical experiences [32, 33]. Specifically, visual exposure to nature has been shown to facilitate recovery, relieve stress and anxiety, decrease physiological arousal by lowering blood pressure, and improve mood [34, 35]. Natural landscapes rapidly capture attention and require less cognitive resources compared to other types of stimuli, thereby promoting psychological well-being and physiological relaxation, as reflected by increased parasympathetic activity [36]. These findings resonate with the naturalistic audiovisual stimuli integrated in our video intervention and reinforce their potential for improving individual well-being during WB-MRI. This beneficial effect has also been highlighted during diagnostic procedures by Sjölander and colleagues (2019), finding that the exposure to nature films during a colonoscopy was associated with reductions in cortisol release, increases in prolactin levels, and improved oxygen saturation, collectively enhancing patients’ subjective experience of the examination [37].

The greater effectiveness of audiovisual entertainment becomes particularly evident when anxiety levels are evaluated using clinical classifications. As previously illustrated in Fig. 4, the proportion of patients in the Video group exhibited clinically severe anxiety before the WB-MRI exam significantly decreased from 33.8% pre-procedure to just 12.7% after the exam. A similar, albeit less pronounced, reduction was observed in the Music group, where clinically severe anxiety declined from 22.5% to 12.7% after the DWB-MRI examination. Conversely, the Standard group exhibited a slight increase in the percentage of patients with clinically severe anxiety, rising from 14.1% before to 16.3% after the exam. These findings strongly support the effectiveness of both interventions, with the Video group showing the most substantial anxiolytic effect. Consistent results have been reported by Shimokawa et al. (2022), who observed that patients with a high pre-MRI anxiety undergoing MRI with an audiovisual system experienced a greater anxiety relief compared to those receiving standard care [38].

A similar pattern was observed for claustrophobia levels reported by patients, which significantly contributed to the observed variations in anxiety during the DWB-MRI examination. Our results highlighted that participants with severe claustrophobia exhibited a distinct trajectory of anxiety reduction during WB-MRI compared to those with mild/moderate claustrophobia. Specifically, individuals with higher baseline anxiety levels and severe claustrophobia experienced a more pronounced decrease in anxiety throughout the procedure. This trend was observable across all experimental groups, but was significantly amplified in participants receiving WB-MRI with video entertainment, implying that visual assistance may be particularly effective in attenuating anxiety during WB-MRI in participants with severe claustrophobia.

These results are consistent with prior studies demonstrating that the implementation of an audiovisual system significantly facilitated the completion of MRI scans in claustrophobic patients by reducing the interruptions and the need for sedation [14].

While these findings are encouraging and suggest significant clinical utility, some limitations should be acknowledged. Although randomized controlled trials are considered the gold standard in study design, we prioritized the feasibility of performing MRI scans in routine clinical practice, which led us to adopt a quasi-experimental design. Additionally, while anxiety was assessed using validated instruments, the findings may be influenced by patients’ subjective perceptions. Future studies would benefit from incorporating objective physiological measures to complement and validate patient-reported outcomes. Additionally, the study was conducted at a single specialized oncology center with a relatively homogeneous patient population, which may affect the generalizability of the findings to other clinical settings or patient groups with different characteristics.

## Conclusion

These findings support the adoption of a patient-centred audiovisual system as a practical strategy to optimise the clinical experience of the procedure and improve the tolerability of patients undergoing WB-MRI. The implementation of these entertainment systems not only enhances individuals’ subjective experience but could also confers tangible clinical and economic benefits, such as reducing scan duration, minimizing motion artifacts, and improving image quality. Furthermore, audiovisual distraction has the potential to lower sedation requirements and potentially mitigate the development of long-term phobic responses frequently associated with MRI procedures [14].

Improving WB-MRI tolerability may also foster greater adherence to screening programs, thereby facilitating earlier malignancy detection and improving prognostic outcomes. This is critically important in oncology, where timely diagnosis underpins effective treatment and survival. Ultimately, overcoming barriers to WB-MRI acceptance supports broader integration of this technique into routine clinical workflows, advancing healthcare equity and promoting population health through a personalized medicine framework [39].

## Supplementary Information

Below is the link to the electronic supplementary material.

Supplementary Material 1

## Data Availability

The datasets generated and/or analyzed during the current study are available from the corresponding author on reasonable request.

## Abbreviations

WB-MRI: Whole-body magnetic resonance imaging
IRCCS: Hospitalization and Healthcare
IEO: European Institute of Oncology
FLAIR: Axial fluid-attenuated inversion recovery
DT: Distress Thermometer
STAI-Y: State-Trait Anxiety Inventory subscale
MRI-AQ: MRI-anxiety questionnaire
IQR: Interquartile range
